# 55P0110, a Novel Synthetic Compound Developed from a Plant Derived Backbone Structure, Shows Promising Anti-Hyperglycaemic Activity in Mice

**DOI:** 10.1371/journal.pone.0126847

**Published:** 2015-05-14

**Authors:** Barbara Brunmair, Zsuzsanna Lehner, Karin Stadlbauer, Immanuel Adorjan, Klaus Frobel, Thomas Scherer, Anton Luger, Leonhardt Bauer, Clemens Fürnsinn

**Affiliations:** 1 Division of Endocrinology & Metabolism, Department of Medicine III, Medical University of Vienna, Vienna, Austria; 2 55pharma Drug Discovery & Development AG, Vienna, Austria; Baylor College of Medicine, UNITED STATES

## Abstract

Starting off with a structure derived from the natural compound multiflorine, a derivatisation program aimed at the discovery and initial characterisation of novel compounds with antidiabetic potential. Design and discovery of the structures was guided by oral bioactivities obtained in oral glucose tolerance tests in mice. 55P0110, one among several new compounds with distinct anti-hyperglycaemic activity, was further examined to characterise its pharmacology and mode of action. Whereas a single oral dose of 55P0110 did not affect basal glycaemia, it markedly improved the glucose tolerance of healthy and diabetic mice (peak blood glucose in glucose tolerance test, mmol/l: healthy mice with 90 mg/kg 55P0110, 17.0±1.2 vs. 10.1±1.1; diabetic mice with 180 mg/kg 55P0110, 23.1±0.9 vs. 11.1±1.4; p<0.001 each). Closer examination argued against retarded glucose resorption from the gut, increased glucose excretion in urine, acute insulin-like or insulin sensitising properties, and direct inhibition of dipeptidyl peptidase-4 as the cause of glucose lowering. Hence, 55P0110 seems to act via a target not exploited by any drug presently approved for the treatment of diabetes mellitus. Whereas the insulinotropic sulfonylurea gliclazide (16 mg/kg) distinctly increased the circulating insulin-per-glucose ratio under basal conditions, 55P0110 (90 mg/kg) lacked such an effect (30 min. after dosing, nmol/mol: vehicle, 2.49±0.27; 55P0110, 2.99±0.35; gliclazide, 8.97±0.49; p<0.001 each vs. gliclazide). Under an exogenous glucose challenge, however, 55P0110 increased this ratio to the same extent as gliclazide (20 min. after glucose feeding: vehicle, 2.53±0.41; 55P0110, 3.80±0.46; gliclazide, 3.99±0.26; p<0.05 each vs. vehicle). By augmenting the glucose stimulated increase in plasma insulin, 55P0110 thus shows distinct anti-hyperglycaemic action in combination with low risk for fasting hypoglycaemia in mice. In summary, we have discovered a novel class of fully synthetic substituted quinazolidines with an attractive pharmacological profile that recommends the structures for further evaluation as candidates for the treatment of diabetes mellitus.

## Introduction

Despite technological progress, a considerable number of drugs reaching the market are still directly or indirectly derived from natural products. This likewise applies to new chemical entities approved for the treatment of diabetes mellitus and includes formally synthetic compounds that can be traced back to a scaffold of natural origin serving as the initial lead for development [1,2]. Against this background, traditional herbal remedies used for diabetes treatment may serve as a source of chemical lead structures for the design of superior synthetic analogues.

In the traditional ethnic medicine of the Mediterranean area, lupins are used for the treatment of diabetes mellitus. Their presumptive antidiabetic activity has been ascribed to quinozolidine alkaloids, which are abundant in these plants. Such compounds have previously been associated with glucose lowering effects in experimental rodent models as well as with direct insulinotropic effects on pancreatic islets [3–5]. Accordingly, feeding diabetic animals with extracts from *Lupinus termis* or *Medicago sativa*, which contain lupin alkaloids, has been described to ameliorate hyperglycaemia, hypercholesterinemia and DNA damage [6–8]. Kubo et al. focussed on one among the pharmacologically active lupin alkaloids, (-)-multiflorine, and demonstrated that a single dose acutely improves glucose tolerance in mice. Based on this finding, they successfully designed several derivatives of (-)-multiflorine with robust glucose lowering activities [9,10]. We have now undertaken an effort to use one of these structures as a blueprint for a more extensive derivatisation program directed towards the discovery and development of novel antidiabetic agents.

At present, most attempts in drug discovery emanate from a pre-defined molecular target. They proceed by identification and optimisation of chemical structures based on their direct interaction with this target in vitro. In contrast to this reductionistic approach, our starting point was a compound with documented glucose lowering activity in vivo, but with an unknown molecular target and mechanism of action. Consequently, our strategy for structural optimisation relied on a highly disease relevant readout for oral bioactivity (a standardised oral glucose tolerance test in mice) rather than on information about direct interaction of two chemical structures in an artificial environment. Our approach did not only ascertain activity in the live organism. It also allowed to consider additional relevant parameters for drug development like distinctive acute side effects, solubility, formulation properties and bioavailability at the earliest possible stage. The search for effective analogues was accompanied by a stepwise elaboration of the underlying mode of action. As a result of the here described endeavour, we report the discovery, development and initial pharmacological characterisation of several highly effective antidiabetic structures, and we provide first information about the mechanism responsible for their glucose lowering activity.

## Materials and Methods

### Animal husbandry

Male healthy C57BL/6J and obese diabetic db/db mice were purchased from Charles River Laboratories (Sulzfeld, Germany). Mice were housed in polycarbonate cages provided with wood-based bedding (Hygienic Animal Bedding, J.Rettenmaier & Söhne, Rosenberg, Germany) under constant room temperature and an artificial 12 h dark/12 h light cycle. Unless stated otherwise, they had free access to tap water and conventional chow diet (sniff R/M-H; sniff Spezialdiäten GmbH; Soest, Germany).

All experimental procedures followed the principles of good laboratory animal care and were in line with effective national and international guidelines and law. The protocols were approved by the Austrian Federal Ministry of Science and Research after review by an expert committee authorised by the ministry, as well as by an ethical committee of the Medical University of Vienna (approvals # BMWF-66.009/0190-C/GT/2007 and BMWF-66.009/0074-II/10b/2008).

### Glucose tolerance tests

Novel compounds were synthesised at 55pharma’s chemistry labs (Tulln, Austria) and were tested as racemates (diastereomeric pure). Design and discovery of novel structures was guided by oral bioactivities and structure-activity relationship arising from standardised oral glucose tolerance tests (OGTTs) in mice. Standard OGTTs were performed in male C57BL/6J mice, which were within an age range of 8 to 20 weeks. The groups of mice examined in the same experimental run were weight-matched, and each run included its own vehicle treated control group for direct comparison. Mice were fasted for 10 h, before the tip of the tail was pricked with a needle for the measurement of blood glucose. Immediately thereafter, mice received by gavage the respective test compound or plain vehicle solution (0.5% sodium carboxymethylcellulose, 5 ml/kg body weight). Fortyfive min after dosing, mice were gavaged with aqueous glucose solution (3 g/kg; volume: 6 ml/kg body weight). The resulting excursion of blood glucose was documented by measurements immediately before (0 min) as well as 30, 60, 90, 120, and 150 min after glucose administration. Unless negative immediate effects of the treatment or impaired weight gain were noted, individual mice were used repeatedly for up to 8 experiments in total. Wash out/recovery periods of at least 7 days were scheduled between consecutive tests.

Selected compounds were further characterised in glucose tolerance tests with protocols that deviated from the above-described standard OGTT procedure (differences in mouse strain, time course, glucose dose, and route of administration). Details on these protocol variations are indicated at the corresponding passages of the Results & Discussion section.

### Insulin tolerance test

Food was withdrawn from 14 weeks-old male db/db mice 1 h before the test compound was administered by gavage. 1.5 U/kg insulin was injected intraperitoneally 45 min later (Actrapid, Novo Nordisk, Bagsvaerd, Denmark, diluted with saline; injected volume, 3 ml/kg body weight). Blood glucose was measured immediately before insulin injection (0 min), and 30, 60, 90, and 120 min thereafter.

### Analytics

Blood glucose concentration was measured immediately in duplicate with a portable glucose meter (OneTouch, LifeScan, Milpitas, CA, USA). In OGTTs on db/db mice, blood glucose usually rose above the measurement limit of the portable glucose meter. In these experiments, blood glucose was therefore measured with a photometric assay (Human liquiUV, Wiesbaden, Germany). Plasma insulin was measured with Mouse Ultrasensitive Insulin ELISA from Mercodia (Uppsala, Sweden). Direct effects of compounds on the activity of dipeptidyl peptidase-4 (DPP-4) in vitro were determined with the DPPIV Drug Discovery Kit from tebu-bio (Offenbach, Germany).

### Statistical procedures

Results are given as means±SEM. p-Values were calculated in the context of an exploratory data analysis using two-tailed one-sample or unpaired t-tests with a p<0.05 considered as significant.

## Results and Discussion

### The starting structure 55P0001

**Fig 1:**
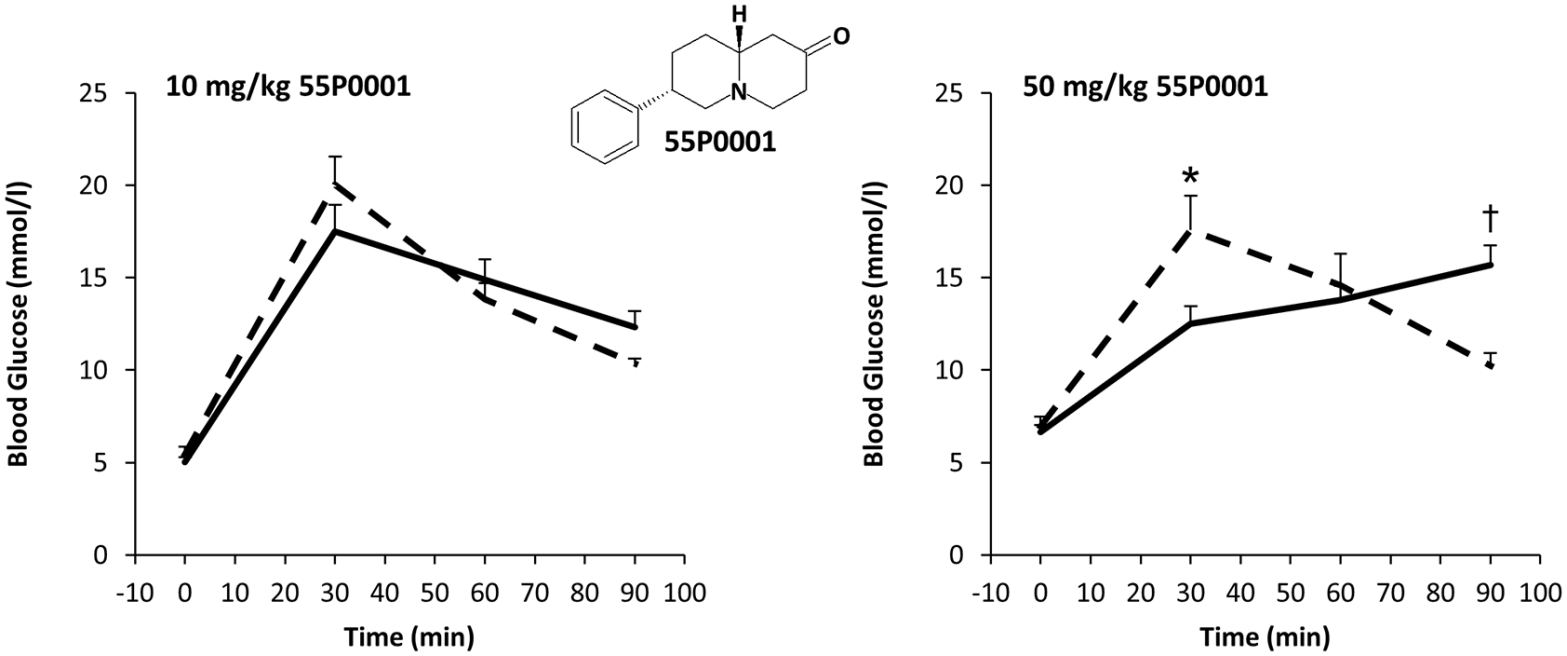
55P0001 initially reduces, but later elevates blood glucose during a glucose tolerance test in mice. Male C57BL/6J mice were intraperitoneally injected with 10 mg/kg (left) or 50 mg/kg (right) 55P0001 (solid lines), or with the vehicle (broken lines) immediately before an oral glucose tolerance test (3 g/kg). The insert shows the chemical structure of 55P0001. Means±SEM; n = 11 each; *p<0.05, †p<0.001 vs. vehicle.

Starting point of the derivatisation program was compound 55P0001, which had been described by others earlier [9]. To confirm and reproduce the previously published results, we performed a GTT exactly along the protocol used by others. Hence, healthy male C57BL mice were subjected to an OGTT (3 g/kg) immediately after intraperitoneal injection of 55P0001. In agreement with the previous report, 55P0001 had a dual effect on glucose tolerance with glucose lowering action during the first part of the OGTT (obvious at 30 min), but elevation of blood glucose thereafter (obvious at 90 min). Both, the rapid glucose lowering component and the delayed glucose rising component, were dose dependent with non-significant trends observed in response to 10 mg/kg 55P0001 (p = 0.24 and p = 0.05 at 30 and 90 min, respectively; Fig 1A), but marked and significant differences after administration of 50 mg/kg 55P0001 (Fig 1B).

### Discovery and bioactivities of selected derivatives

**Fig 2:**
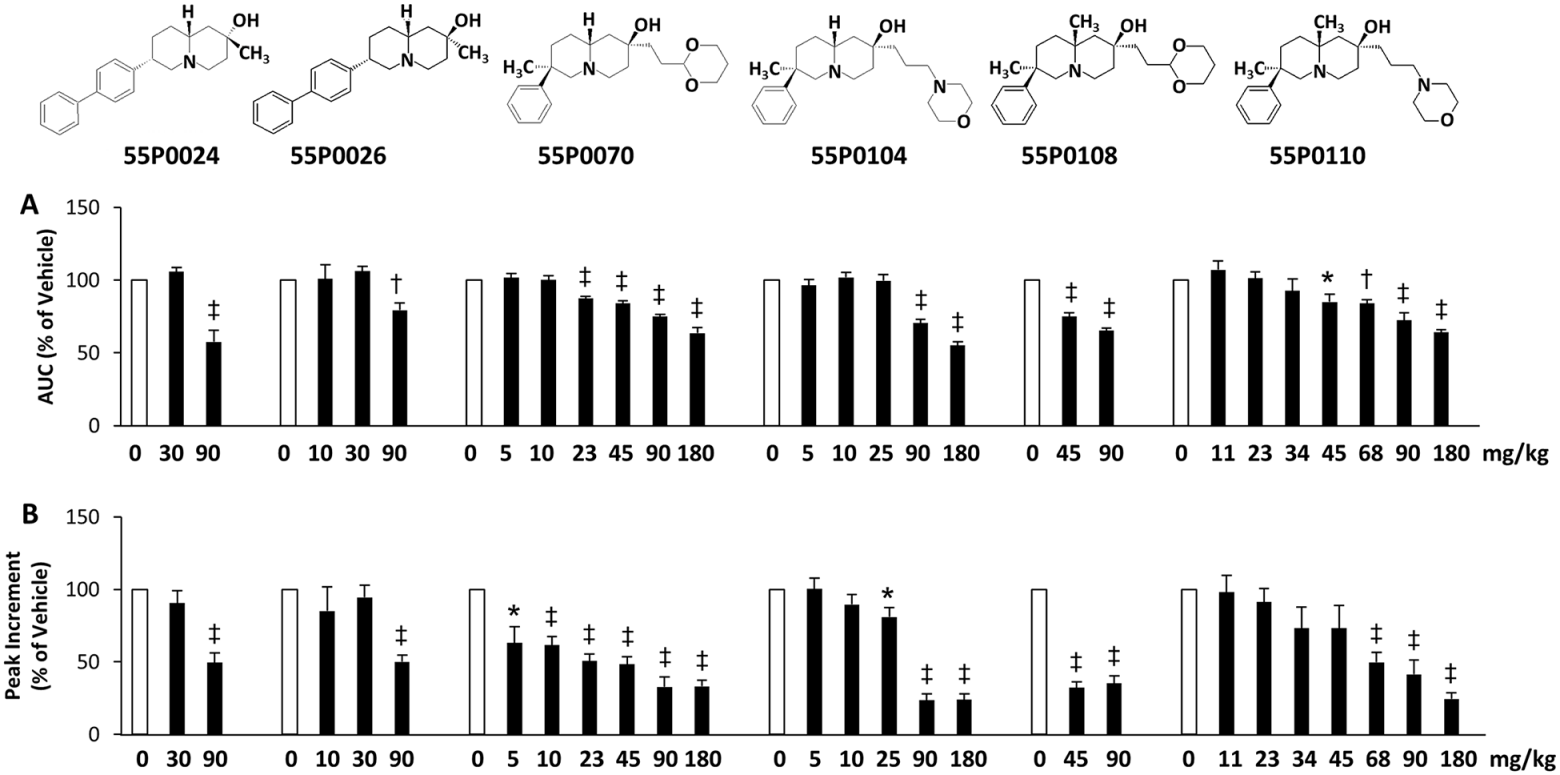
Novel compounds that dose-dependently improve glucose tolerance in mice. The indicated doses of novel compounds (codes and chemical structures shown above the bar graphs) were orally administered to male C57BL/6J mice 45 min before a standard oral glucose tolerance test was started (3 g/kg). Effects on the total area under the glucose curve (AUC; **A**), and on the peak increment in blood glucose 30 min after glucose administration (**B**) are given as % of mean values of the corresponding vehicle group. Means±SEM; n = 6–16 each; *p<0.05; †p<0.01; ‡p<0.001 vs. vehicle (= 100%; open bars).

Based on a stepwise procedure guided by the oral bioactivities of novel derivatives and by interpretation of the arising structure-activity-relationship, numerous substituted quinazolidines were designed, synthesised and examined in the standard OGTT (initial test always at a dose of 90 mg/kg). This strategy led to the discovery of several structures with robust glucose lowering activities, which were neither associated with noticeable acute adverse effects, nor with delayed hyperglycaemia as induced by the starting compound 55P0001 (Fig 1). Fig 2 exemplifies the anti-hyperglycaemic effects of selected structures with such attributes (55P0024, 55P0026, 55P0070, 55P0104, 55P0108, and 55P0110). When tested in the standard OGTT, oral administration of these compounds dose dependently blunted the total area under the glucose curve (Fig 2A) as well as the maximal increment of blood glucose seen at 30 min (Fig 2B).

### Characterisation of 55P0110 in healthy mice

**Fig 3:**
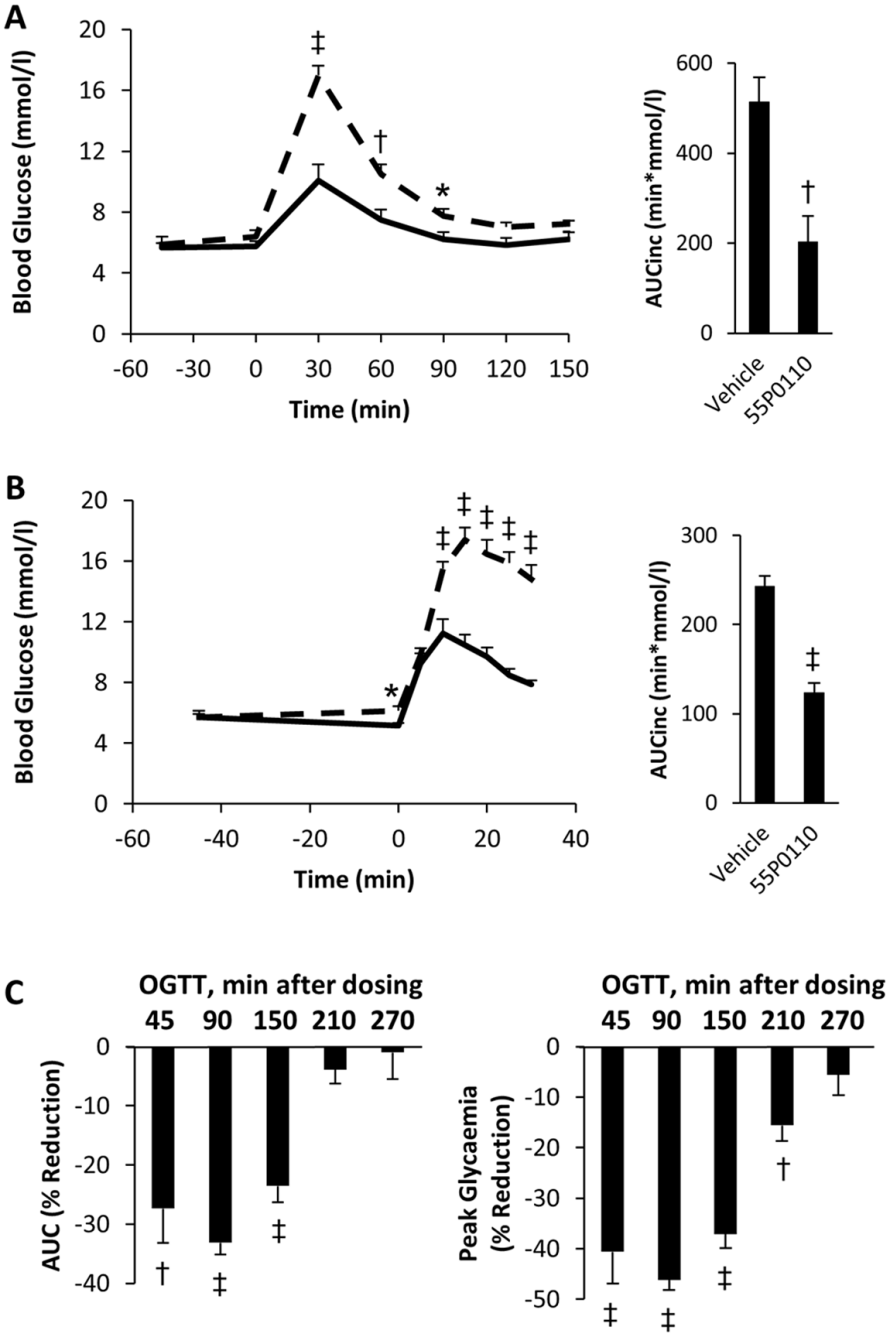
Improvement of glucose tolerance by a single oral dose of 55P0110 in mice. Effects of oral administration of 90 mg/kg 55P0110 (full lines) or vehicle (broken lines) 45 min before starting a glucose tolerance test in male C57BL/6J mice. (**A**) Glucose curve and incremental area under the glucose curve (AUCinc) in the standard oral glucose tolerance test. (**B**) Glucose excursion and AUCinc during the initial 30 min after glucose administration. (**C**) Reductions of total areas under the curves (AUC; left bar graph) and peak blood glucose (30 min after glucose administration; right bar graph) at different time intervals after the administration of 55P0110 (data given in % of mean of the corresponding vehicle treated control group). Means±SEM; n = 8–9 each; *p<0.05; †p<0.01; ‡p<0.001 vs. vehicle.

From the available set of structures with distinct glucose lowering properties, 55P0110 was selected to further explore the pharmacological attributes of a member of this novel compound class. The selection was based on chemical attributes regarded as advantageous for drug development, like high solubility and steric stability. Fig 3A depicts the full curve of the standard OGTT under treatment with 90 mg/kg 55P0110. The results indicate only modest, if any, effect of the drug on basal glycaemia during the initial 45 min after oral administration, but distinct anti-hyperglycaemic action after glucose feeding, as highlighted by a 60% reduction of the incremental area under the glucose curve (p = 0.001). Such a combination of marginal influence on basal glycaemia, but potent counteraction of the increment following glucose ingestion, seems attractive for an antidiabetic drug. It suggests a low risk for hypoglycaemic events, which frequently occur with sulfonylureas or glinides and complicate optimal glycaemic control [11,12].

Fig 3B shows the effect of 55P0110 on glucose excursion during the first 30 min of the standard OGTT. During the initial 5 min after glucose feeding, blood glucose rose to a similar extent in mice treated with 55P0110 and vehicle, but anti-hyperglycaemic action of 55P0110 became very distinct within the subsequent 5 min. Next, we performed standard OGTTs at increasing time intervals after dosing of the drug. Significantly blunted glucose excursion still persisted more than 200 min after oral intake (Fig 3C). Hence, the anti-hyperglycaemic effects of a single oral shot of 90 mg/kg 55P0110 is characterised by rapid albeit not immediate onset, and by a reasonable durability of action for more than 3 h.

**Fig 4:**
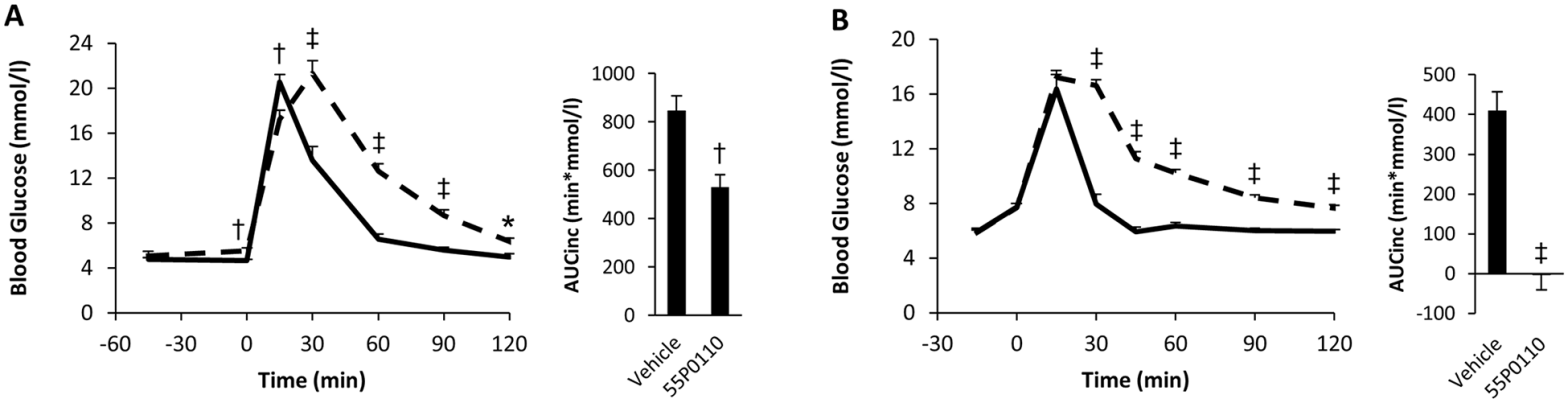
Improvement of glucose tolerance by 55P0110 is independent of the route of administration. Effects of 90 mg/kg 55P0110 (full lines) as compared to vehicle (broken lines) on glucose excursion and incremental area under the glucose curve (AUCinc) in intraperitoneal glucose tolerance tests (2 g/kg) in male C57BL/6J mice. (**A**) Glucose injected 45 min after oral administration of 55P0110. (**B**) Glucose injected 15 min after intraperitoneal injection of 55P0110. Means±SEM; n = 7–9 each; *p<0.05; †p<0.01; ‡p<0.001 vs. vehicle.

The unaffected increase in blood glucose during the initial 5 min after glucose feeding argues against impaired intestinal glucose resorption as the primary mechanism responsible for the anti-hyperglycaemic action of 55P0110. For further clarification, 55P0110 was fed by gavage 45 min before glucose was administered in a parenteral fashion by intraperitoneal injection (2 g/kg). The anti-hyperglycaemic activity of 55P0110 was preserved, which corroborated that inhibition of intestinal glucose resorption is not relevant to the glucose lowering action of 55P0110 (Fig 4A). 55P0110 likewise retained its activity after intraperitoneal injection of both, drug and glucose, which further excludes onset of action within the gut (Fig 4B).

Increased urinary glucose excretion is another mechanism for the lowering of blood glucose, which is exploited by the rising drug class of sodium-glucose cotransporter 2 (SGLT2) inhibitors, the gliflozines [13,14]. We therefore examined, whether the anti-hyperglycaemic activity of our compounds was associated with increased glucosuria. Whenever a mouse treated with 55P0104, 55P0108 or 55P0110 urinated spontaneously during handling for glucose measurement at time points 30 or 60 min of a standard OGTT, we checked for urine glucose content with semiquantitative test strips. Of 28 vehicle treated mice, 3 showed modest urine glucose content (up to 500 mg/dl) and 2 showed even more distinct glucosuria. Glucose was likewise occasionally found in urine from 14 mice treated with doses of 55P0104, 55P0108 or 55P0110, which were too low to affect glycaemia (5 mice with modest and 1 with robust glucosuria). In contrast, we did not observe glucose in urine from any of the 10 mice that had been treated with drug doses causing significant improvement of glucose tolerance. Hence, glucosuria was occasionally seen in the absence of effective glucose lowering treatment, which suggests that the renal threshold for glucose resorption can be exceeded under these conditions. But we never found glucose in the urine of mice, in which the excursion of blood glucose was blunted by one of our compounds. This excludes urine glucose excretion as a relevant anti-hyperglycaemic mechanism of our novel quinazolidines.

### Characterisation of 55P0110 in diabetic mice

**Fig 5:**
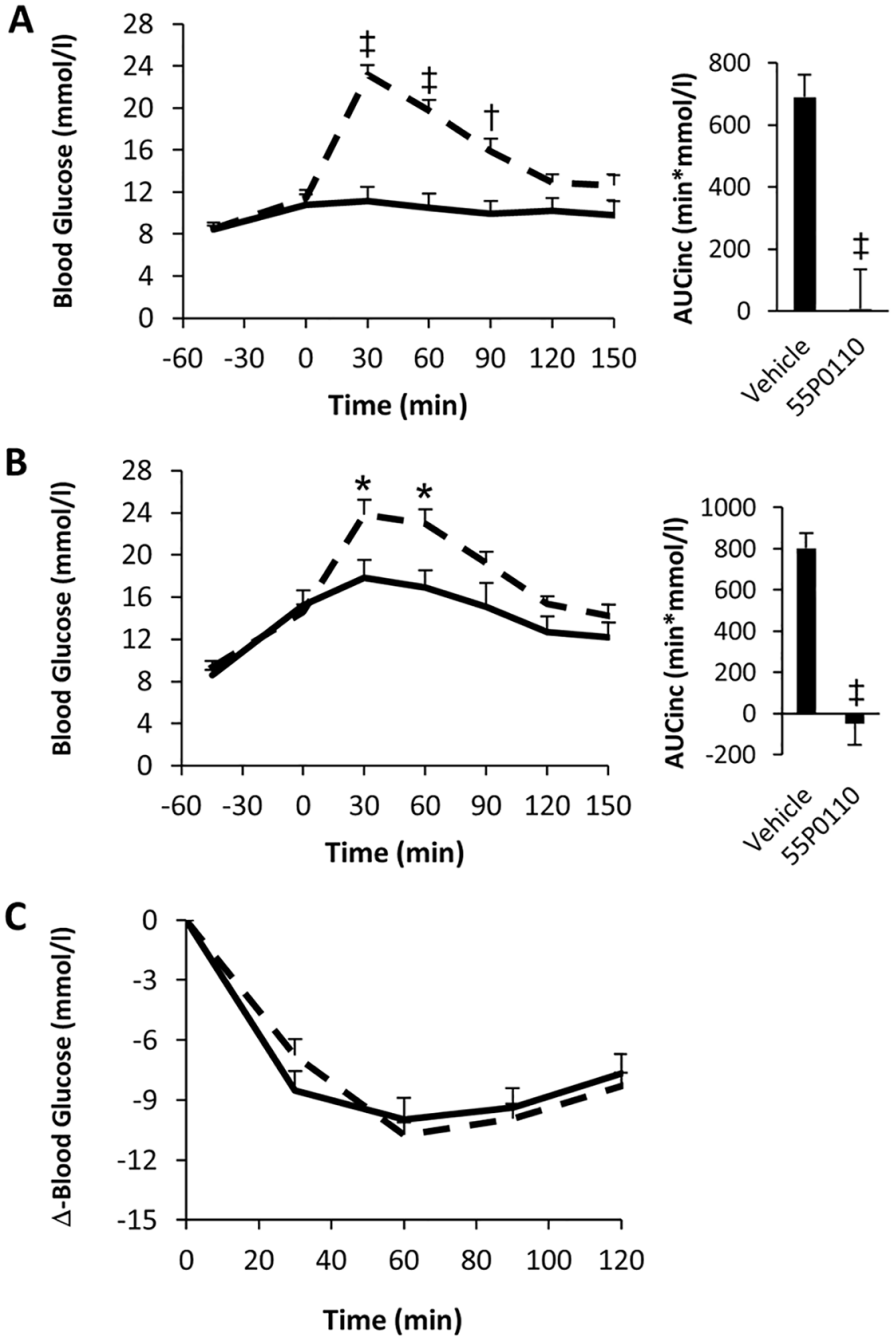
A single dose of 55P0110 improves glucose tolerance but not insulin sensitivity in diabetic mice. Effect of feeding 180 mg/kg 55P0110 to 9 weeks-old (**A**) or 90 mg/kg 55P0110 to 12 weeks-old (**B**) fasted male db/db mice at -45 min before an oral glucose tolerance test (2 g/kg). Glucose curve and incremental area under the glucose curve (AUCinc) are shown. (**C**) Effect of feeding 90 mg/kg 55P0110 at -45 min on an insulin tolerance test (1.5 U/kg) in 14 weeks-old male db/db mice. 55P0110, full lines; vehicle, broken lines; means±SEM; n = 9–10 each; *p<0.05; †p<0.01; ‡p<0.001 vs. vehicle.

The effects of 180 and 90 mg/kg 55P0110 on glucose tolerance were examined also in fasted male db/db mice. To avoid overshooting hyperglycaemia, the amount of glucose fed was lower in OGTTs with diabetic db/db mice than in the standard OGTT procedure used for healthy mice (2 g/kg instead of 3 g/kg). 55P0110´s ability to powerfully counteract the rise of blood glucose during an OGTT was fully preserved in hyperglycaemic db/db mice, which is highlighted by incremental glucose areas that were close to zero (Fig 5A and 5B). It is also of note that the endogenous rise of basal blood glucose seen 45 min after the first gavage (a well-known consequence of handling-induced arousal in diabetic mice) remained unaffected by 55P0110. These data suggest that 55P0110, in spite of distinct effects on the fate of exogenous glucose, does not impair physiological mechanisms that account for an endogenous rise of blood glucose under stress conditions. Furthermore, the dependence on the exogenous origin of glucose, as seen in lean as well as in obese diabetic mice, obviously excludes insulin-like action as the anti-hyperglycaemic mechanism of 55P0110.

The insulin resistant db/db mice were also exposed to an insulin tolerance test. The observed insulin induced fall in blood glucose remained unaffected by a preceding dose of 55P0110 (Fig 5C). This result clearly argued against acute modulation of insulin sensitivity as the cause of glucose lowering by 55P0110. Furthermore, lack of differences between controls and treated animals during the late insulin tolerance test suggests an unimpaired counter-regulatory response to hypoglycaemia. This desirable attribute of 55P0110 adds to the above-mentioned impression of no interference with endogenous mechanisms for raising blood glucose.

### Comparison to established drugs

**Fig 6:**
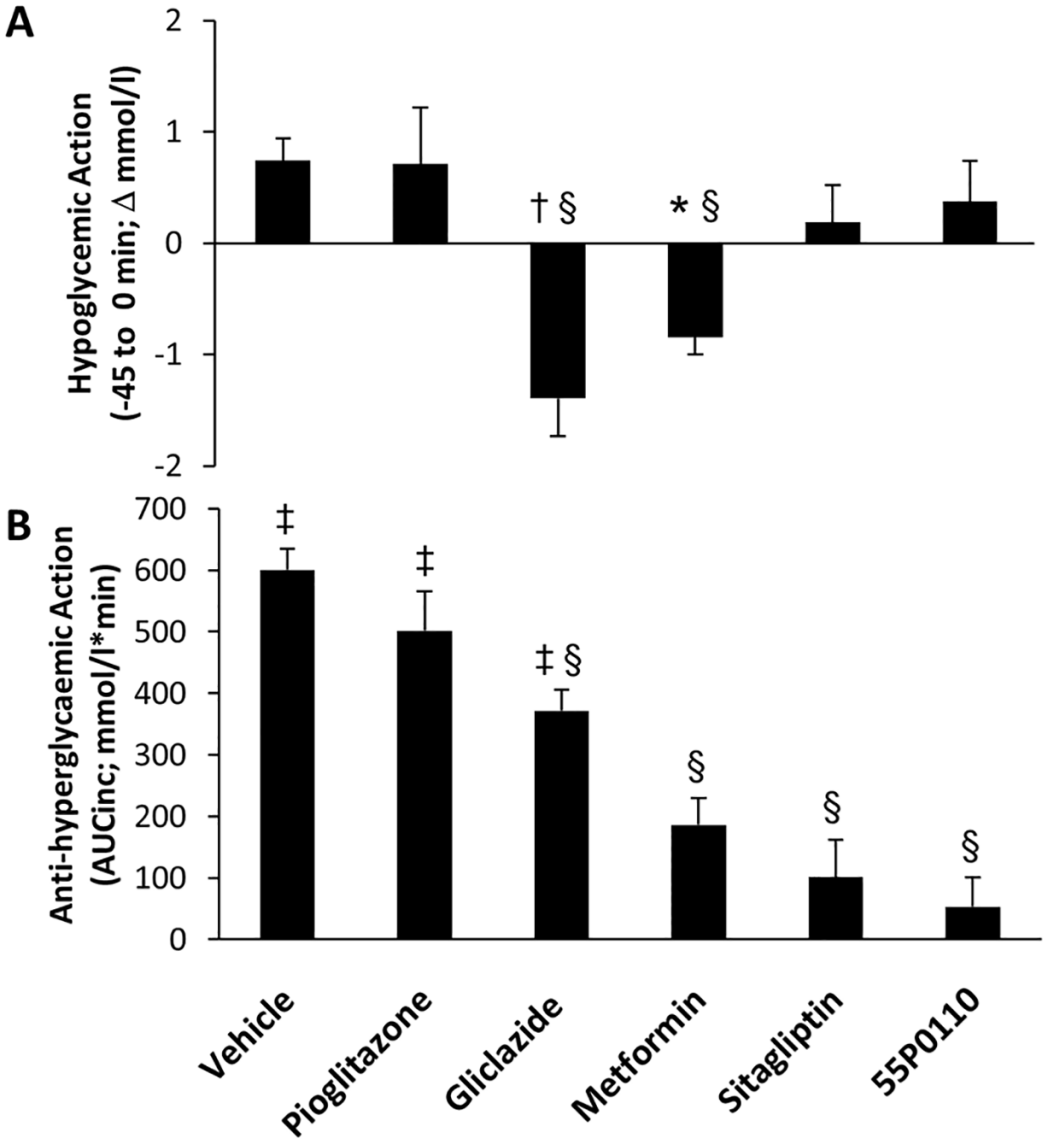
Comparison of 55P0110 to established anti-diabetic drugs—hypoglycaemic and anti-hyperglycaemic properties in mice. Vehicle, pioglitazone (30 mg/kg), gliclazide (8 mg/kg), metformin (200 mg/kg), sitagliptin (10 mg/kg), or 55P0110 (90 mg/kg) were orally administered to mice 45 min before a standard oral glucose tolerance test was started (3 g/kg). (**A**) Change in basal glycaemia 45 min after drug feeding („hypoglycaemic action“); (**B**) incremental area under the glucose curve (AUCinc; „anti-hyperglycaemic action“). Means±SEM; n = 8–27 each; *p<0.05; †p<0.01; ‡p<0.001 vs. 55P0110; §p<0.05 vs. vehicle.

After the exclusion of several mechanisms of action, 55P0110 was further characterised in our standard OGTT procedure by head-to-head comparison to oral antidiabetic drugs that belong to different clinically established drug classes. The doses chosen for the various compounds were in the high range of those frequently used in mouse experiments by others [15–19]. Full glucose excursion curves are shown in S1 Fig. The most crucial findings are pinpointed in Fig 6, which compares the drugs with regard to their unwanted hypoglycaemic activity (changes in basal glycaemia 45 min after drug feeding; Fig 6A) as well as with regard to their desirable anti-hyperglycaemic activity (incremental areas under the glucose curves; Fig 6B).

As our setting portrays the acute effect of a single dose, pioglitazone lacked any effect on glucose tolerance, which is in line with need for regular pioglitazone treatment to induce insulin sensitisation. The biguanide metformin and the sulfonylurea gliclazide showed robust hypoglycaemic as well as anti-hyperglycaemic action, although metformin fell somewhat behind gliclazide with regard to the magnitude of the respective responses. At first sight, a sulfonylurea-like pattern of metformin action appears unexpected, but the acute response to a single metformin dose in rodents must be distinguished from the delayed effects of regular treatment as relevant to patients. The typical feature of 55P0110, *i*.*e*. hardly any hypoglycaemic effect accompanied by distinct anti-hyperglycaemic action, was only shared by sitagliptin, which lowers blood glucose via direct inhibition of DPP-4 [20]. Comparison of increasing doses of 55P0110 and sitagliptin in our standard OGTT further corroborated the impression of notable similarities between their effects (S2 Fig). These observations evidently led to the question about possible parallels in their mechanisms of action.

**Fig 7:**
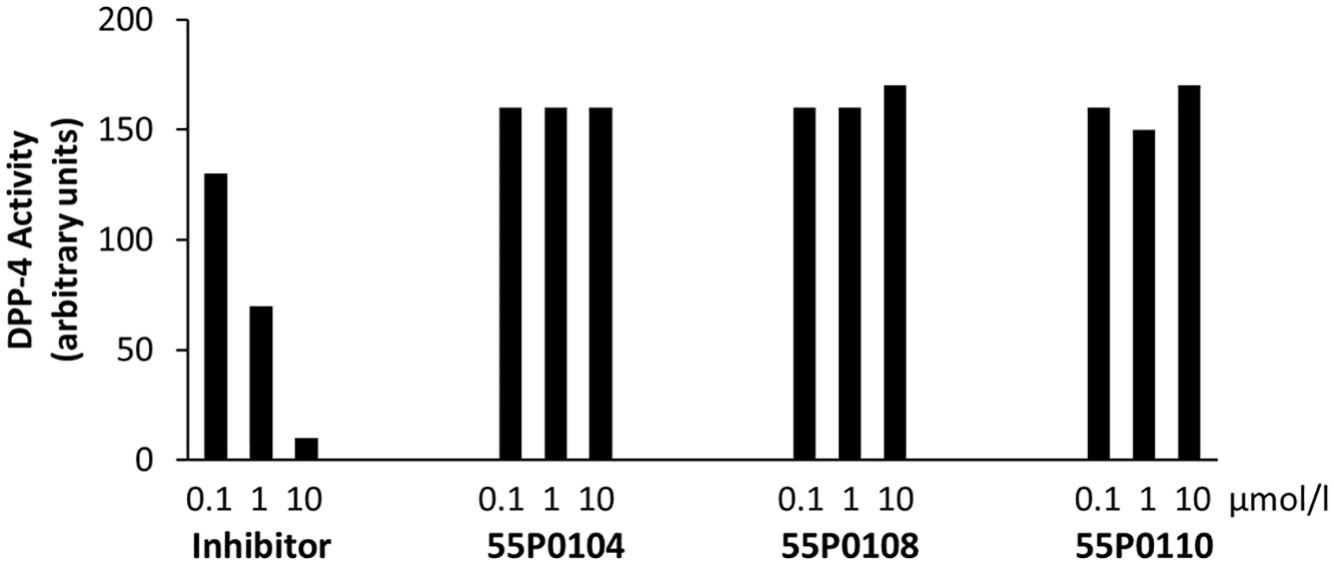
No direct effect of novel compounds on DPP-4 activity in vitro. Direct effects of 0.1, 1, or 10 μmol/l 55P0104, 55P0108, 55P0110, or an established DPP-4 inhibitor as provided by the assay manufacturer on DPP-4 activity in vitro. Substrate conversion measured at least in duplicate at 5 min intervals over 60 min.

Hence, we next examined if 55P0110, like the gliptins, is a direct inhibitor of DPP-4. Using a DPP-4 activity assay, we tested some of our novel compounds at concentrations up to 10 μmol/l, which is above the plasma levels found in pharmacokinetic pilot experiments on mice fed 100 mg/kg 55P0110 (S1 Table). The DPP-4 activity assay did not unmask any inhibitory effect of 55P0110, 55P0104 or 55P0108, which excluded this enzyme as their direct molecular target (Fig 7).

Still, the striking similarities between sitagliptin and 55P0110 seen in the OGTT could result from a common mode of action downstream of divergent initial target structures. Inhibition of DPP-4 by gliptins augments insulin release in a glucose dependent manner, which is mediated by an increment in circulating glucagon-like peptide (GLP-1) [20,21]. It could therefore be that 55P0110, like sitagliptin, acts by potentiating glucose stimulated insulin release, even if not via DPP-4 inhibition. To sort this out, we determined insulin-per-glucose ratios in blood from 55P0110 treated and gliclazide treated mice under basal conditions as well as after oral glucose administration.

**Fig 8:**
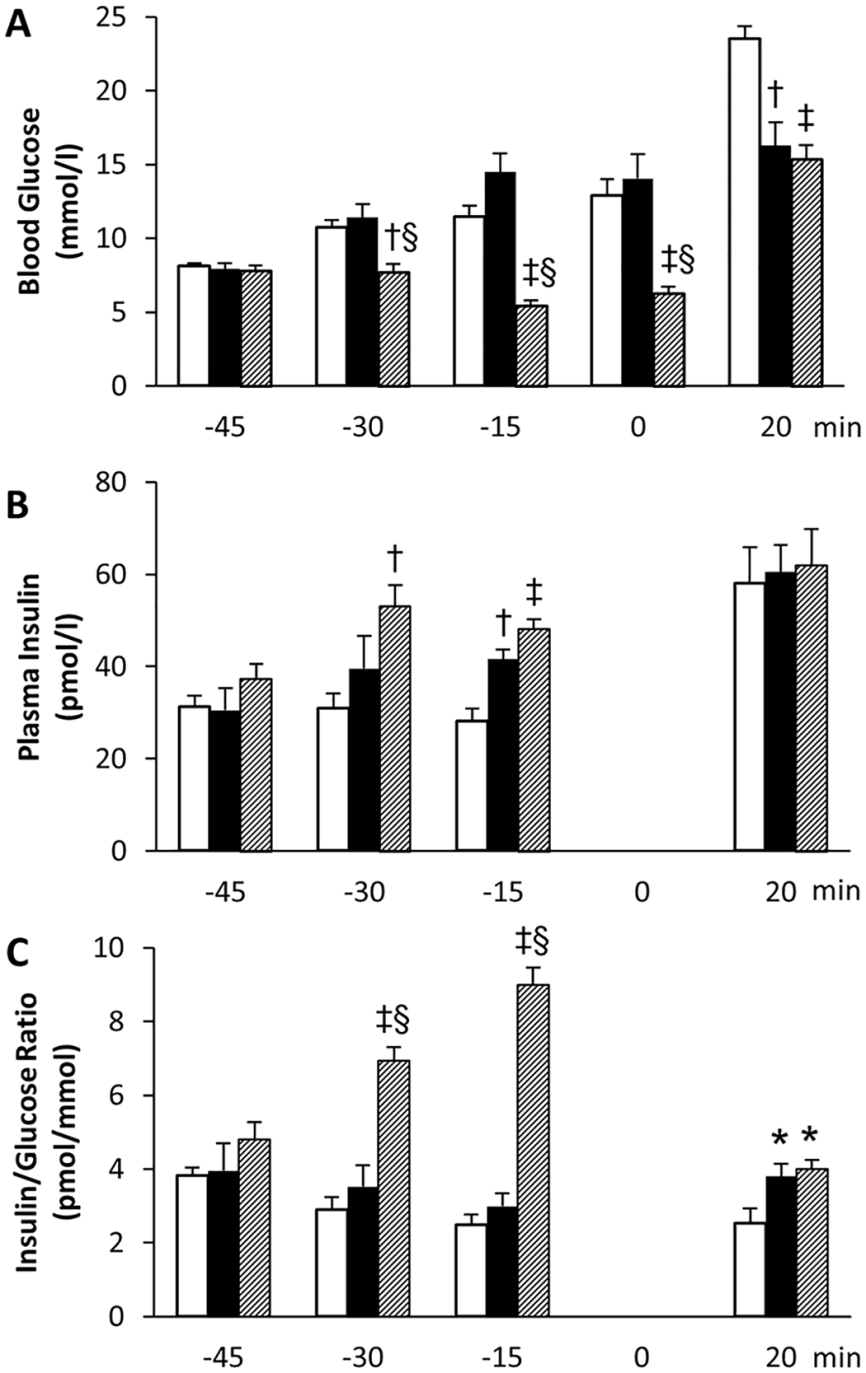
At variance to gliclazide, 55P0110 raises the circulating insulin-per-glucose ratio only under hyperglycaemic conditions. The effects of oral administration at -45 min of vehicle (white bars), 90 mg/kg 55P0110 (black bars), or 16 mg/kg gliclazide (hatched bars) on blood glucose (**A**), plasma insulin (**B**), and the insulin-per-glucose ratio (**C**) in male C57BL/6J mice before and after feeding glucose (3 g/kg at 0 min). Means±SEM; n = 5–6 each; *p<0.05; †p<0.01; ‡p<0.001 vs. vehicle; § p<0.05 for gliclazide vs. 55P0110.

Under basal conditions, *i*.*e*. before the administration of exogenous glucose, the insulin-per-glucose ratio remained completely unaffected by 55P0110 (an increase in basal plasma insulin observed after 30 min could be attributed to slightly higher glycaemia, p = 0.08; Fig 8). In contrast to this, basal plasma insulin increased while glycaemia decreased immediately after the administration of gliclazide. This resulted in almost 3-fold higher insulin-per-glucose ratios in mice receiving gliclazide than such treated with vehicle or 55P0110. Although gliclazide shows less unwanted hypoglycaemic potential than other sulfonylureas in the clinic [22], the response obtained in mice obviously portrays the well-known attribute of this drug class to retain full insulinotropic action in the presence of low glucose [21].

However, the picture changed as soon as exogenous glucose was fed. Albeit plasma insulin was in the same range in all mice 20 min after glucose dosing (including those treated with the vehicle), blood glucose was blunted to a similar extent in mice treated with 55P0110 and gliclazide. Consequently, the similar anti-hyperglycaemic effects of the employed doses of 55P0110 and gliclazide were accompanied by similar increases in the insulin-per-glucose ratios (Fig 8). The results thus suggest that both drugs augment insulin release under glucose stimulated conditions, whereas only the sulfonylurea does so under basal conditions.

## Conclusions

Our resumption of preceding endeavours to develop orally bioactive derivatives of (-)-multiflorine thus led to the discovery of a novel class of fully synthetic substituted quinazolidines with blood glucose lowering properties in mice. Initial pharmacological characterisation focussed on the immediate effects of a single oral dose of compound 55P0110. Despite harbouring a low potential for hypoglycaemia, 55P0110 outmatched most established oral antidiabetic drugs in a mouse OGTT. Our experiments suggest that 55P0110 acts via a mechanism different from those exploited by all oral drugs, which are presently approved for the clinical treatment of diabetes mellitus. A direct molecular target has not yet been identified, but our results imply that the mechanism of 55P0110-induced glucose lowering relies on the augmentation of glucose stimulated insulin release. This fits with published pilot experiments about the direct effects of multiflorine on isolated pancreatic islets [5]. Potentiation of glucose-induced insulin secretion has been associated with several receptors on pancreatic ß-cells (e.g. GLP-1 and GIP-receptors, and GPR40). Hence, the next challenge will be to investigate, whether the action of our new compounds involves effects on these receptors and/or on their endogenous agonists. In summary, the distinct glucose lowering potential, the low risk for fasting hypoglycaemia, and the attractive pharmacological profile arising from our experiments on mice recommend the discovered structures for further evaluation as potential antidiabetic drugs.

## Supporting Information

S1 Fig Comparison of effects of oral antidiabetic drugs on glucose tolerance in mice. 55P0110 (90 mg/kg; A), sitagliptin (10 mg/kg; B), gliclazide (8 mg/kg; C), pioglitazone or metformin (30 and 200 mg/kg; D) were orally administered to male C57BL/6J mice 45 min before a standard oral glucose tolerance test was started (3 g/kg). Means±SEM; 8–16 each; *p<0.05; †p<0.01; ‡p<0.001 vs. vehicle.
(PDF)

S2 Fig Dose-dependent effects of 55P0110 and sitagliptin on glucose tolerance in mice. 55P0110 (45, 90, 180 mg/kg; A) or sitagliptin (1, 3, 30 mg/kg; B) were orally administered to male C57BL/6J mice 45 min before a standard oral glucose tolerance test was started (3 g/kg). Means±SEM; n = 7–9 each.
(PDF)

S1 Table Pharmacokinetic pilot data from mice treated with 100 mg/kg 55P0110. At 0 min, male C57BL/6J mice received by gavage an oral dose of 100 mg/kg 55P0110. One mouse was killed for blood sampling at each indicated time interval after drug administration.
(PDF)

S2 Table Compilation of original data. This file provides an Excel compilation of all original raw data that have been used in the manuscript.
(XLSX)
