# Supplementary material for: 55P0110, a Novel Synthetic Compound Developed from a Plant Derived Backbone Structure, Shows Promising Anti-Hyperglycaemic Activity in Mice
Source: PLoS One. 2015 May 14;10(5):e0126847. doi: 10.1371/journal.pone.0126847 (PMC4431753; doi:10.1371/journal.pone.0126847)
Supplement: S1 Fig — 55P0110 (90 mg/kg; A), sitagliptin (10 mg/kg; B), gliclazide (8 mg/kg; C), pioglitazone or metformin (30 and 200 mg/kg; D) were orally administered to male C57BL/6J mice 45 min before a standard oral glucose tolerance test was started (3 g/kg). Means±SEM; 8–16 each; *p<0.05; †p<0.01; ‡p<0.001 vs. vehicle. (PDF) [file pone.0126847.s001.pdf]

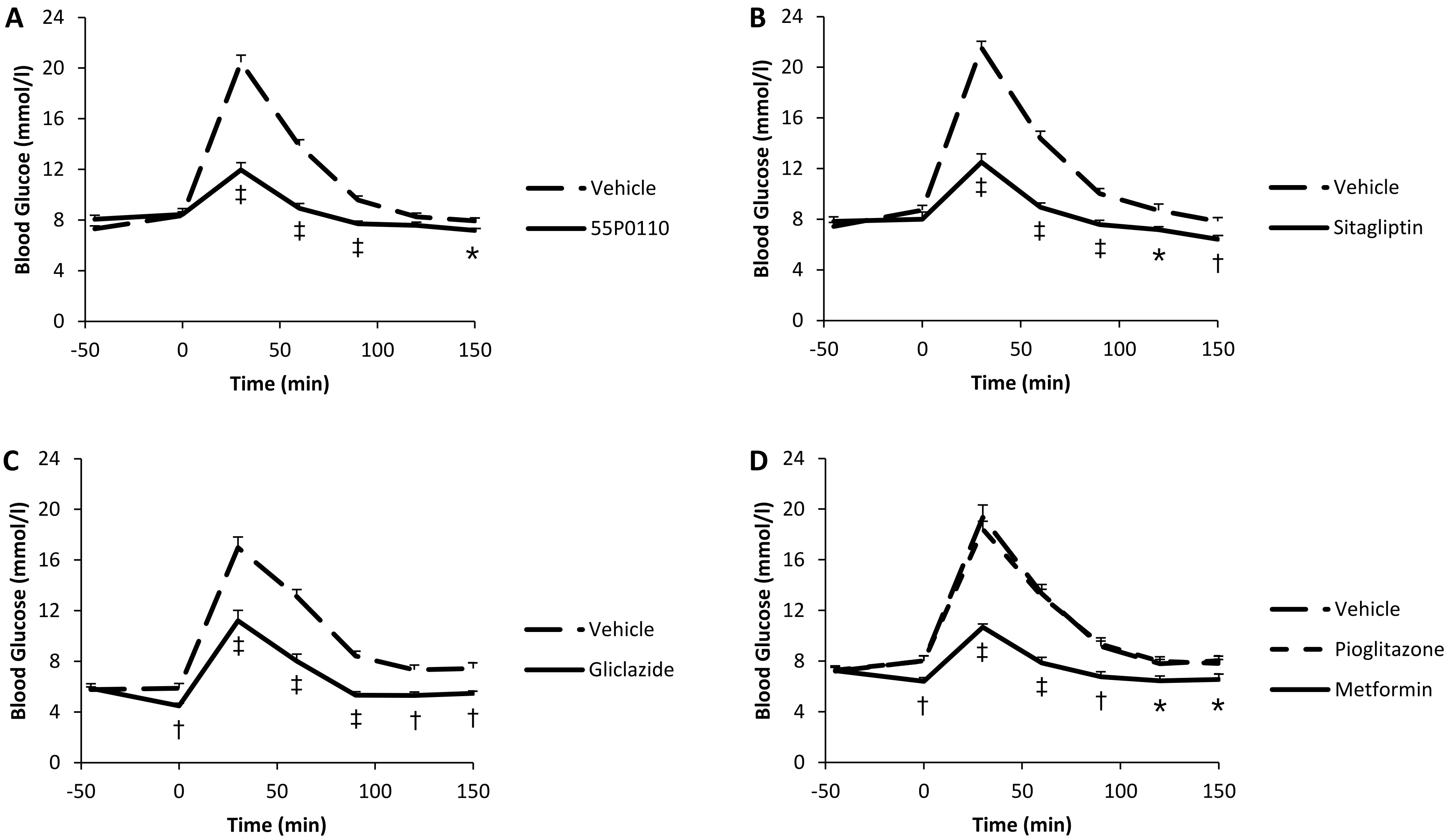

<u>S1 Fig.</u> Comparison of effects of oral antidiabetic drugs on glucose tolerance in mice. 55P0110 (90 mg/kg; **A**), sitagliptin (10 mg/kg; **B**), gliclazide (8 mg/kg; **C**), pioglitazone or metformin (30 and 200 mg/kg; **D**) were orally administered to male C57BL/6J mice 45 min before a standard oral glucose tolerance test was started (3 g/kg). Means±SEM; 8-16 each; *p<0.05; †p<0.01; ‡p<0.001 vs. vehicle.
