# Supplementary material for: 55P0110, a Novel Synthetic Compound Developed from a Plant Derived Backbone Structure, Shows Promising Anti-Hyperglycaemic Activity in Mice
Source: PLoS One. 2015 May 14;10(5):e0126847. doi: 10.1371/journal.pone.0126847 (PMC4431753; doi:10.1371/journal.pone.0126847)
Supplement: S2 Fig — 55P0110 (45, 90, 180 mg/kg; A) or sitagliptin (1, 3, 30 mg/kg; B) were orally administered to male C57BL/6J mice 45 min before a standard oral glucose tolerance test was started (3 g/kg). Means±SEM; n = 7–9 each. (PDF) [file pone.0126847.s002.pdf]

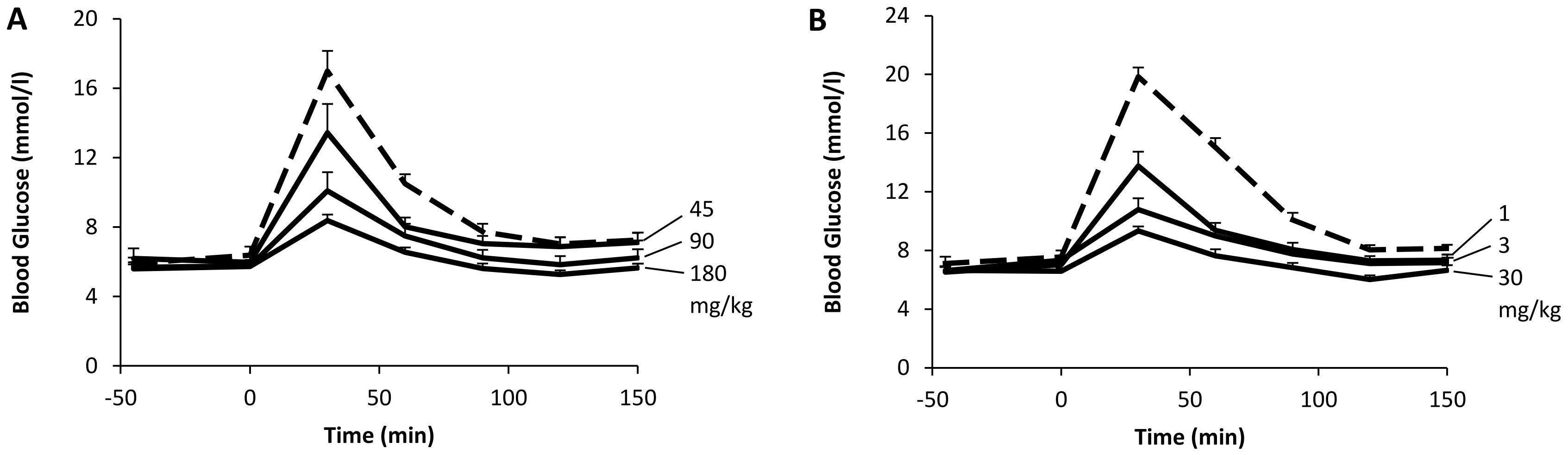

<u>S2 Fig.</u> Dose-dependent effects of 55P0110 and sitagliptin on glucose tolerance in mice. 55P0110 (45, 90, 180 mg/kg; **A**) or sitagliptin (1, 3, 30 mg/kg; **B**) were orally administered to male C57BL/6J mice 45 min before a standard oral glucose tolerance test was started (3 g/kg). Means±SEM; n=7-9 each.
