# Supplementary material for: 55P0110, a Novel Synthetic Compound Developed from a Plant Derived Backbone Structure, Shows Promising Anti-Hyperglycaemic Activity in Mice
Source: PLoS One. 2015 May 14;10(5):e0126847. doi: 10.1371/journal.pone.0126847 (PMC4431753; doi:10.1371/journal.pone.0126847)
Supplement: S1 Table — At 0 min, male C57BL/6J mice received by gavage an oral dose of 100 mg/kg 55P0110. One mouse was killed for blood sampling at each indicated time interval after drug administration. (PDF) [file pone.0126847.s003.pdf]

<u>S3 Table.</u> Pharmacokinetic pilot data from mice treated with 100 mg/kg 55P0110.

At 0 min, male C57BL/6J mice received by gavage an oral dose of 100 mg/kg 55P0110. One mouse was killed for blood sampling at each indicated time interval after drug administration.

Time after Dosing (min)	Plasma 55P0110 (µmol/l)
0	0.00
7.5	1.99
15	6.19
30	4.07
60	4.30
90	6.32
180	1.68
